# Clinical and genomic features in patients with second primary glioblastoma following first primary renal cell carcinoma

**DOI:** 10.1186/s12885-023-10541-x

**Published:** 2023-01-30

**Authors:** Guang-Tao Zhang, Qi Liu, Fu-Xing Zuo, Hou-Jie Liu, Song-Quan Wang, Qing Yuan, Ang-Si Liu, Ke Hu, Xiao-Li Meng, Wei-Jia Wang, Hai-Peng Qian, Jing-Hai Wan, Hong-Qing Cai

**Affiliations:** 1 Department of Neurosurgery, National Cancer Center/National Clinical Research Center for Cancer/Cancer Hospital, Chinese Academy of Medical Sciences and Peking Union Medical College, Beijing, 100021 China; 2 Department of Neurosurgery, The Second Affiliated Hospital, Anhui Medical University, Hefei, 230601 China; 3 State Key Laboratory of Molecular Oncology, Center for Cancer Precision Medicine, National Cancer Center/ National Clinical Research Center for Cancer/Cancer Hospital, Chinese Academy of Medical Sciences and Peking Union Medical College, Beijing, China

**Keywords:** Glioblastoma, Renal cell carcinoma, Clinical feature, Genomic feature

## Abstract

**Purpose:**

To explore the potential pathogenesis and clinical features of second primary glioblastoma (spGBM) following first primary renal cell carcinoma (fpRCC).

**Methods:**

Patients with spGBM after fpRCC were enrolled from our institution and the SEER dataset. Sanger sequencing, whole genome sequencing, and immunehistochemistry were used to detect molecular biomarkers.

**Results:**

Four and 122 cases from our institution and the SEER dataset, respectively, were collected with an overall median age of 69 years at spGBM diagnosis following fpRCC. The median interval time between fpRCC and spGBM was 50.7 months and 4 years, for the four and 122 cases respectively. The median overall survival time was 11.2 and 6.0 months for the two datasets. In addition, spGBM patients of younger age (< 75 years) or shorter interval time (< 1 year) had favorable prognosis (*p* = 0.081 and 0.05, respectively). Moreover, the spGBM cases were molecularly classified as *TERT* only paired with *TP53* mutation, *PIK3CA* mutation, *EGFR* alteration, low tumor mutation burden, and stable microsatellite status.

**Conclusions:**

This is the first study to investigate the pathogenesis and clinical features of spGBM following spRCC. We found that spGBMs are old-age related, highly malignant, and have short survival time. Moreover, they might be misdiagnosed and treated as brain metastases from RCC. Thus, the incidence of spGBMs after fpRCC is underestimated. Further studies are needed to investigate the underlying molecular mechanisms and clinical biomarkers for the development of spGBM following fpRCC.

## Introduction

In the past decades, the treatment of cancer, including radiotherapy, chemotherapy, and target therapy has undergone solid advances, and the rate of tumor control and patient survival time has markedly improved. Thus, there has been an increasing incidence of secondary primary malignancy in patients with prolonged survival time. Moreover, genetic predisposition, irradiation, and systematic therapeutic agents may individually or synergistically contribute to the development of a second primary malignancy. Apparently, patients with a new secondary primary malignancy are different from those with a single malignant tumor. They have more confusing clinical features and require a more sophisticated treatment strategy.

Studies on second primary GBMs (spGBMs) are very rare. Because they have similar neuroimaging findings and non-specific symptoms of intracranial hypertension to the brain metastases of the first primary tumor, they are extremely easily misdiagnosed as brain metastases in clinical practice. Previous studies reported that they occurred in acute lymphocytic leukemia (ALL) patients with a 10 to 20 times greater risk than age-matched healthy controls [1]. Moreover, radiation used to control hematological malignancy and primary CNS tumors can induce the occurrence of GBMs [2]. In addition, it has also been reported that second primary GBM occurs in Lynch syndrome families with germline mutations in MLH1, MSH2, MSH6, and PMS2, and Turcot syndrome families with APC gene mutation [3].

In this study, we report on spGBMs following first primary renal cell carcinoma (fpRCC) and to the best of our knowledge, this has not yet been published. Moreover, we explored the clinical characteristics and genomic features of spGBMs. Our study may increase the understanding of these diseases and promote the improvement of clinical diagnosis and treatment.

## Methods

### Patient selection

The Ethics Committee of Cancer Hospital, Chinese Academy of Medical Sciences approved this retrospective study (No.NCC2014G-12). Written informed consent for sampling and research was obtained from all patients. Twenty-three previously diagnosed RCC patients with neuroimaging diagnosis of single solid tumors in the brain were identified to meet the surgical indications at the neurosurgery department between July 2013 and May 2021. All resected tumor tissues were diagnosed by pathologists according to the morphological characteristics of the tumor cells and their similarity to paired RCC tissues.

Patient information from the SEER database was extracted using SEER*Stat, version 8.3.8. All patients with malignancy located in the central nervous system and urinary system were collected according to the International Classification of Diseases for Oncology Site Recode (third edition, ICD-O-3) rule. The target cases with histopathological diagnosis of RCC followed by GBM were identified based on identical patient numbers.

### Detecting of glioma’s common molecular alterations

Anti-EGFRvIII antibody (working solution, ZA-0643, ZSGB-BIO) and Anti-ATRX antibody (1:500, ab188027, abcam) was used to detect EGFRvIII and ATRX expression. Sanger sequencing for detecting *TERT* promoter mutations and immunohistochemistry (IHC) was performed as described previously [4, 5].

### DNA extraction and whole exon sequencing (WES)

Genomic DNA was isolated from FFPE blocks using a genomic DNA purification kit (Promega), following the manufacturer’s instructions. DNA was quantified using a NanoDrop ND-1000 spectrophotometer (NanoDrop Technologies). Matched DNA from normal renal tissues was used to characterize the genomic alterations in RCC and GBM. Exome capture libraries were prepared in duplicate from the DNA using the SureSelect Human All Exon Human Exome library kit (Agilent) and sequenced on an Illumina HiSeq PE150 platform of BGI (Shenzhen, China).

### Quantification of gene alterations, CNVs, TMB and MSI by WES

The sequencing adapter and low-quality bases were trimmed by fastp (version v0.20.1), the high-quality reads were aligned to human genome(hg19) using Burrows-Wheeler Aligner (bwa, version v0.7.17) with default parameter. Sentieon (version 202010.01) was used to remove duplicate reads, recalibrate base quality score, and call somatic variant with TNhaplotyper command tumor-normal matched mode. subsequently, ANNOVAR (version 20180416) was used to annotate SNVs and Indels, non-synonymous mutations in exonic regions and splice sites are retained. CNVkit (version 0.9.9) was used to call somatic CNVs, all matched normal samples were combined as copy number pooled reference. CBS (Circular binary segmentation) algorithm was used to connect copy number ratio with a similar log2 value to the bin size to obtain information about the change in the number of copies of the segment region. log2 is greater than 0 for gain, and log2 is less than 0 for loss. MSI status was evaluated by Msisensor (version 0.6) with paired mode. all non-synonymous mutations in all exonic regions and splice sites were used to calculate TMB value [6–9].

### Statistical analysis

All statistical analyses were mainly performed in the statistical programming environment R. Patient age was normally distributed, and Student’s t-test was used to compare age differences between groups. The differences in other parameters between the two groups were determined using the Mann–Whitney U test. The “survival” package was used to perform log-rank analysis and draw survival curves. Statistical significance was set at *p* < 0.05. All tests were two-sided.

## Result

### Clinical features of spGBM

**Fig. 1:**
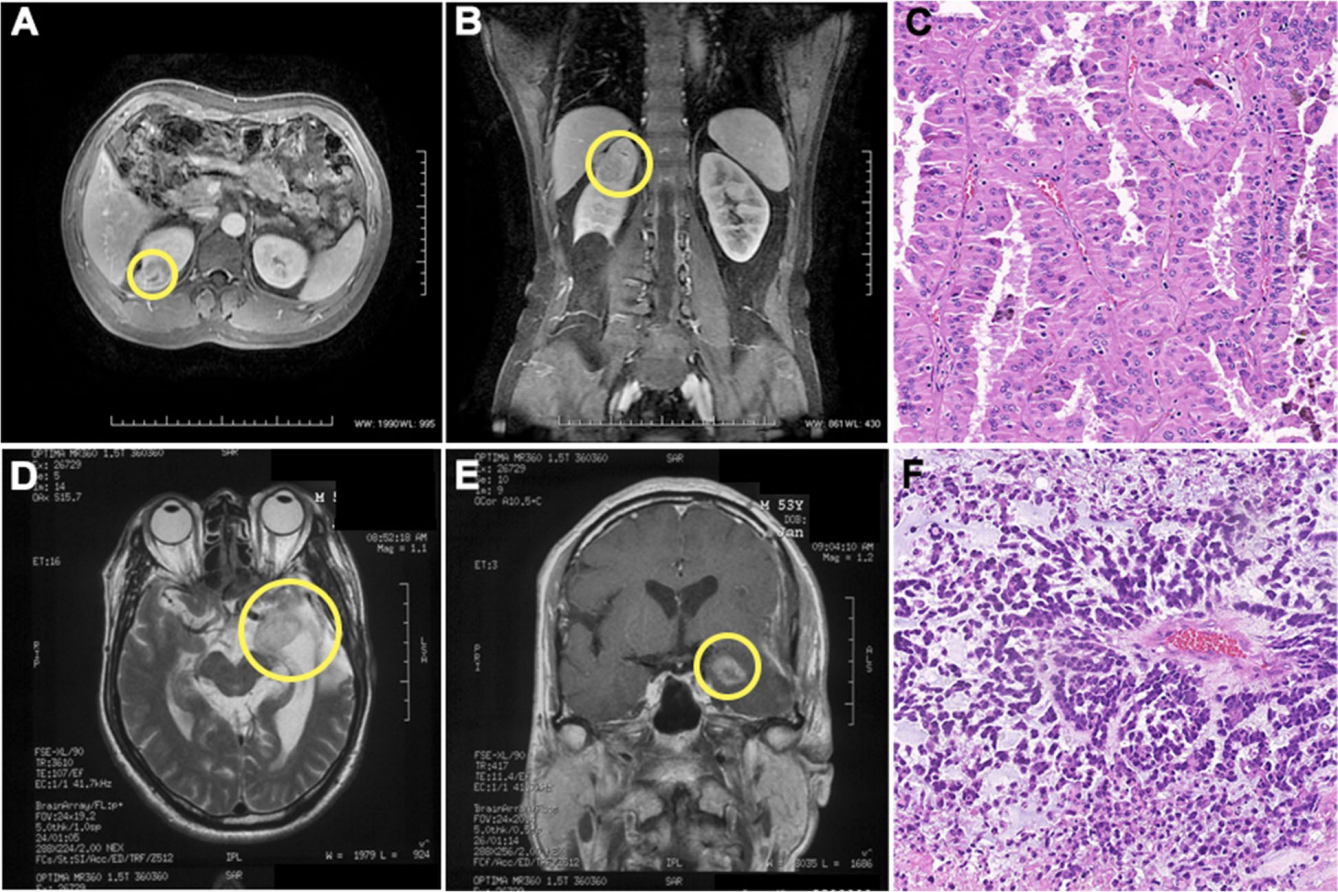
Representative radiographic and histologic appearances of spGBM and fpRCC. **A**-**B** Typical MRI images of fpRCC. **C** HE image of fpRCC. **D**-**E** Typical MRI images of spGBM. **F** HE image of spGBM

Four patients with spGBM following fpRCC were enrolled, including two men and two women. All RCC stages were T1N0M0 according to the 8^th^ American Joint Committee on Cancer staging system. The mean age of all patients was 69 years when diagnosed with spGBM. The median interval time between fpRCC and spGBM was 50.7 months. All patients died of GBM after treatment with the Stupp regimen with a median overall survival time of 11.2 months. The baseline characteristics are listed in detail in Table 1, and representative radiography and histopathology images of the four cases are shown in Fig. 1.

**Table 1:** Baseline information of 4 cases

Case	Gender	Age at RCC	RCC stage	RCC’s Treatment	Interval time (months)	GBM’s treatment	Age at spGBM	Status	Survival time (months)
1	Male	76	T1N0M0	Surgery	47.5	Stupp regimen	80	Dead	11.2
2	Female	66	T1N0M0	Surgery	23.0	Stupp regimen	68	Dead	3.2
3	Female	62	T1N0M0	Surgery + IL-2	92.9	Stupp regimen	70	Dead	15.1
4	Male	49	T1N0M0	Surgery + IL-2	53.9	Stupp regimen^a^	53	Dead	14.2

^a^standard radiotherapy plus concomitant daily temozolomide, followed by adjuvant temozolomide

Moreover, 122 cases (88 men and 34 women) were identified from the SEER dataset according to the inclusion criteria. The median age of all patients was 63.5 (57.0–73.0) and 69.0 (63.0–77.0) years when diagnosed with fpRCC and spGBM, respectively. In total, 82.0% (100/122) and 11.5% (14/122) of fpRCCs were in localized and regional/distant stages, respectively. The median interval time between fpRCC and spGBM from the SEER dataset was 4.0 (1.0–9.0) years. The demographic and clinical characteristics of this cohort are summarized in Table 2.

**Table 2:** Baseline information of enrolled cases from the SEER dataset

Characteristics	No
Gender	
Male	88
Female	34
Age at fpRCC (year)	
< 60	41
> = 60	81
fpRCC stage	
Localized	100
Regional	13
Distant	1
Unstaged	8
Interval time (year)	
> = 1	101
< 1	21
Age at spGBM (year)	
< 60	17
> = 60	105
Death cause	
RCC	13
GBM	86
other	13

### Survival analysis and prognostic factors

**Fig. 2:**
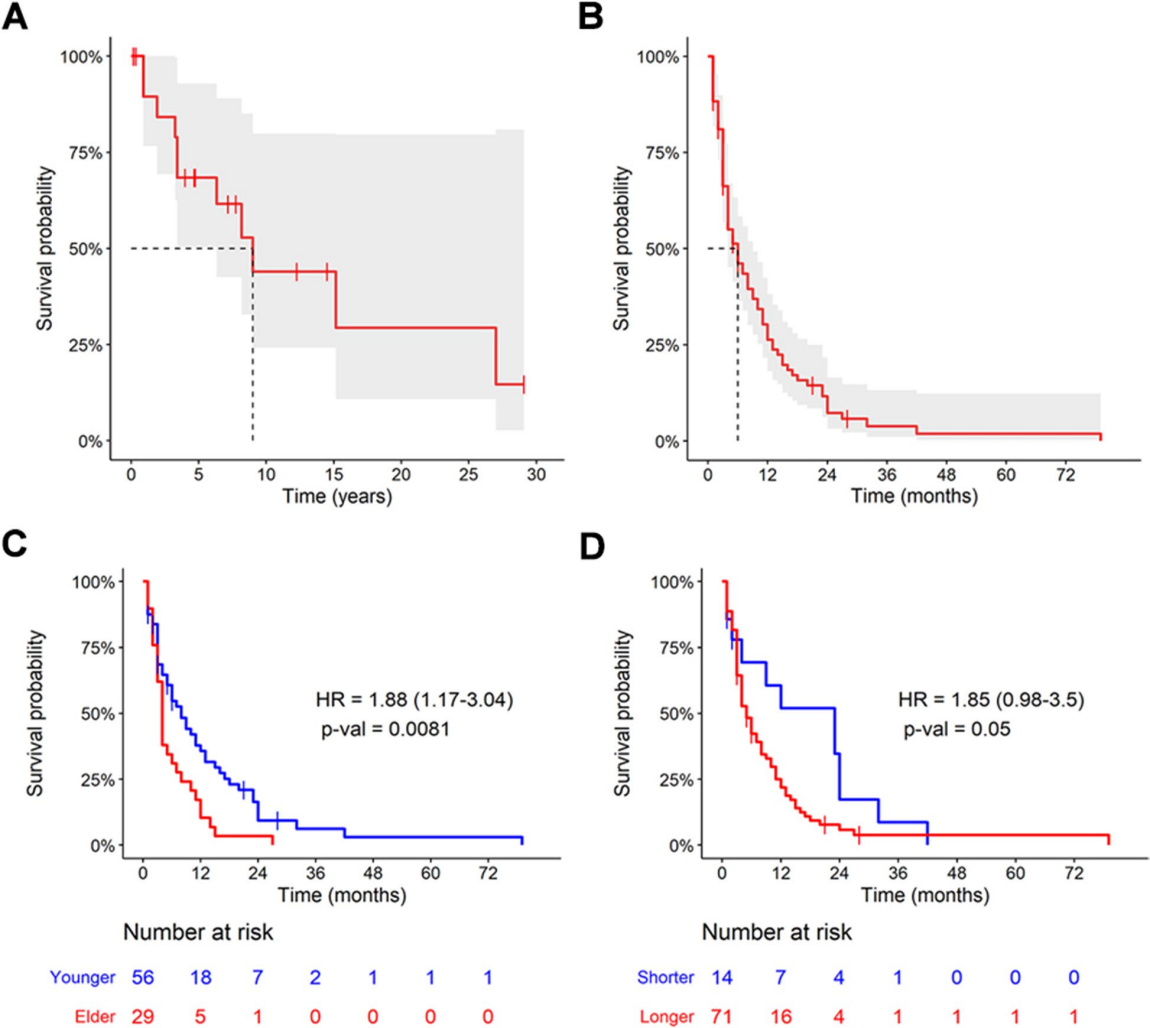
Survival analysis of cases from SEER dataset. **A**-**B** Kaplan–Meier curves of patients with fpRCC and spGBM. **C**-**D** Younger age (< 75 years old) or shorter interval time (< 1 year) indicated favorable prognosis

Of the 112 deceased cases, 13 and 86 died of fpRCC and spGBM, respectively. 13 remaining cases died of other causes. We found that patients who died of fpRCC were younger than those who died of spGBM (mean age: 66.0 vs 70.9 years, *p* = 0.036). However, there were no differences in sex ratio (*p* = 0.927) and in the interval time between fpRCC and spGBM (*p* = 0.338). We performed survival analysis using the K-M method and univariate Cox regression in patients who met the criteria for prognostic analysis (survival time ≥ 1 month). The median survival time were 9 (5.85–not reached) years and 6.0 (4.0–9.0) months for fpRCC and spGBM patients from the SEER dataset, respectively (Fig. 2A-B). In addition, younger spGBM patients (< 75 years old) or with shorter interval time (< 1 year) had favorable prognosis (*p* = 0.0081 and 0.05, respectively) (Fig. 2C-D).

We further performed multivariate Cox regression analysis, and found that interval time was not an independent predictor for prognosis (HR = 1.550, 95% CI: 0.791–3.038, *p* = 0.202). Whereas, age might serve as an independent predictor for prognosis (HR = 1.642 95% CI: 0.997–2.704, *p* = 0.052).

### Genomic profiling of spGBM

**Fig. 3:**
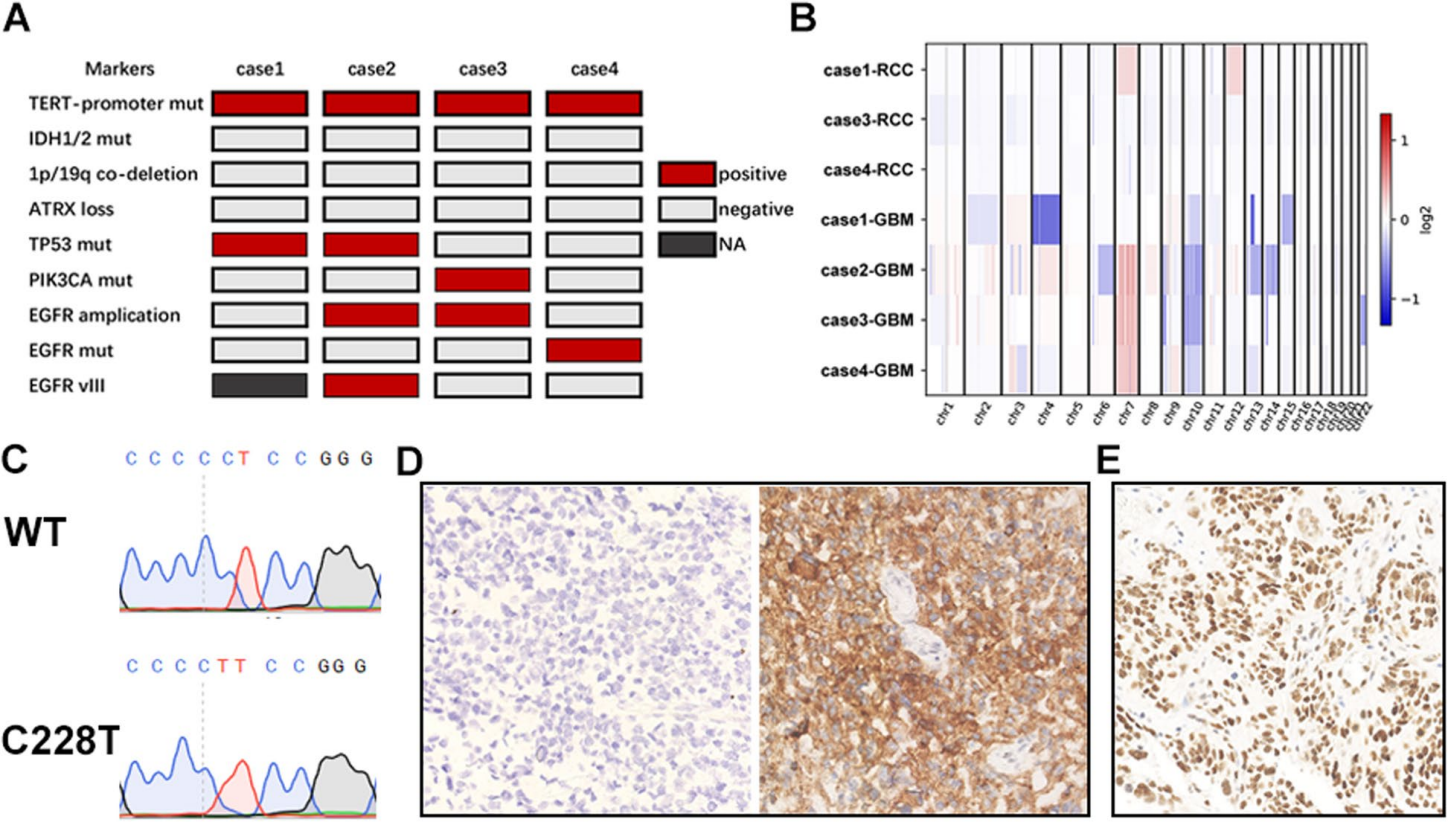
Molecular alterations in spGBM. **A** The molecular findings of 4 sequenced cases. **B** Presentation of CNV profiles for 4 GBMs and 3 RCCs. **C** Sequence chromatograms of wild type and somatic mutations at chr 5: 1,295,228 C > T (C228T) in the TERT promoter locus of case 1. **D**-**E** Representative immunohistochemistry images of EGFRvIII (negative and positive) and ATRX (positive)

Sanger sequencing, whole genome sequencing, and IHC were used to detect the molecular biomarkers of the four cases according to WHO CNS5. We found that all four patients had a *TERT* promoter mutation and no *IDH1/2* mutation, 1p/19q co-deletion, and ATRX loss. Two *TP53* mutations; P33R and R141C, and two *EGFR* amplifications were found. Case 2, which had the shortest survival time, contained both *EGFR* amplification and *EGFR* variant III. In addition, an EGFR extracellular domain mutation, A289D and *PIK3CA* mutation, and E81K, were observed in cases 1 and 3, respectively (Fig. 3). Furthermore, we calculated the tumor mutation burden (TMB) using established gene panels, including F1CDx and MSK-IMPACT. The results showed that case 1, 3, and 4 (case 2 without normal tissue as control) were defined as having low TMB. In addition, the microsatellite status was determined to be stable according to MSISensor score. Detailed analysis results are presented in Table 3.

**Table 3:** The calculated TMB and MSI of 3 cases

Group	TMB		MSI		
	TMB (Muts/MB) (F1CDx^a^)	TMB (Muts/MB) (MSK-IMPACT^b^)	Total number of sites	Number of somatic sites	%
case1-RCC	0.77	0.51	5291	25	0.47
case3-RCC	3.94	2.81	4208	28	0.67
case4-RCC	4.7	3.87	5052	49	0.97
case1-GBM	2.41	1.68	5308	30	0.57
case3-GBM	2.33	1.64	4214	68	1.61
case4-GBM	2.04	1.61	5095	69	1.35

^a^FoundationOne CDx

^b^Memorial Sloan Kettering Cancer Center’s Integrated Mutation Profiling of Actionable Cancer Targets

## Discussion

Imaging findings of parenchymal masses after treatment for malignancies from other body sites often predispose oncologists to make the diagnosis of brain metastasis. This diagnosis is correct in most cases, but may be misdiagnosed by omitting the diagnosis of the primary brain tumor. We conducted a survey of surgical removal of brain tumors after treatment for other malignancies in our medical center over a period of approximately 8 years. We found that primary GBM was the most common type, with an occurrence rate of 17.4% (4/23) among patients who underwent neurosurgery to remove brain tumors and previously diagnosed RCC. Given that a very high percentage of these patients did not undergo brain tumor resection and could not be diagnosed with primary GBM, the incidence of spGBMs after fpRCC is underestimated. In addition, almost all glioma-related clinical trials tend to exclude patients with a history of other non-CNS malignancies. Thus, the potential pathogenesis and clinical features of this type of disease have not been reported and should be elucidated.

Previous studies have shown that the phenotypic development of secondary primary glioma is attributed either to genetic syndrome or therapeutic measures, including pharmacotherapy and cranial radiation therapy [1–3, 10]. In our study, none of the four patients had a treatment history of cranial radiation, and only two cases had a medication history of IL-2. Thus, it is not possible that spGBM following RCC was induced by clinical treatment. Considering that the median age of patients diagnosed with spGBM is approximately 70 years, which is significantly older than the control group (GBM cases from our institution in the same period) [11] and the Chinese Glioma Genome Atlas (CGGA), we are inclined to believe that the accumulated carcinogenic factors significantly increased the risk of GBM in RCC patients. Further analysis would be helpful in investigating the underlying molecular mechanisms and clinical biomarkers for the development of spGBM following fpRCC.

Previous studies by Hamza et al. also found that malignant gliomas were synchronous or metachronous primary non-CNS neoplasms [12]. However, primary RCC was not in the list of non-CNS neoplasms. The main reason may be that patients with history of RCC who develop brain lesions rarely undergo neurosurgery. In addition, they also found that the occurrence of primary non-CNS neoplasm and the duration time did not affect the survival outcome of secondary GBM. Distinctly different, patients with spGBM had poorer prognosis than our control group and other similar aged cases from a previous study [13], and the interval time indicated shorter survival time. Older age may be one reason for the poor prognosis. Moreover, the spGBM cases were molecularly classified as *TERT* only, which was considered to have poorer overall survival according to the molecular scheme [14]. Besides, *TP53* mutation, *PIK3CA* mutation, and *EGFR* alteration make the prognosis worse [15].

As mentioned above, spGBM following fpRCC might be misdiagnosed as brain metastasis from RCC if there is no pathological reference. According to the NCCN recommendations, maximal safe resection is the first-line treatment for primary GBM. Similarly, neurosurgery has been recommended as a rapid and efficient local therapy to resect brain tumors for solitary localized RCC-BM. However, the percentage of patients undergoing neurosurgery is very low in clinical management. Thus, most spGBM patients may miss the opportunity for neurosurgery. In recent years, immune checkpoint inhibitors (ICIs) and tyrosine kinase inhibitors (TKIs) have shown higher intracranial responses than before in clinical trials and are expected to become standard treatment regimens [16, 17]. However, accumulating evidence suggests the limited efficacy of ICIs and TKIs in the treatment of GBM [18]. In addition, our results indicate that spGBMs were in the status of low TMB, stable MSI, and no therapeutic targets also supported the limited efficacy of ICIs and TKIs. Therefore, we strongly recommend neurosurgical treatment for single brain tumors subsequent to RCC.

Our study is a retrospective analysis, and it is unable to standardize the treatment of all patients, especially cases from the SEER dataset. In addition, the small number of sequenced tissues prohibited deep exploration of the pathogenesis of spGBM.

## Conclusion

This is the first study to investigate the pathogenesis and clinical features of spGBMs following fpRCC. We found that spGBMs are old-age related, highly malignant, and have short survival time. Moreover, we propose that the incidence of spGBMs subsequent to fpRCC is underestimated; they might have been misdiagnosed and treated as brain metastases from RCC. Thus, we strongly recommend neurosurgical treatment for single-brain tumors subsequent to RCC. Further studies are needed to investigate the underlying molecular mechanisms and clinical biomarkers for the development of spGBM following fpRCC.

## Data Availability

The datasets generated during and/or analysed during the current study are available from the corresponding author on reasonable request.
